# Mechanisms of Impaired Brown Adipose Tissue Recruitment in Obesity

**DOI:** 10.3389/fphys.2019.00094

**Published:** 2019-02-13

**Authors:** Martín Alcalá, María Calderon-Dominguez, Dolors Serra, Laura Herrero, Marta Viana

**Affiliations:** ^1^Department of Chemistry and Biochemistry, Facultad de Farmacia, Universidad San Pablo-CEU, CEU Universities, Madrid, Spain; ^2^Department of Biochemistry and Physiology, School of Pharmacy, Institut de Biomedicina de la Universitat de Barcelona (IBUB), Universitat de Barcelona, Barcelona, Spain; ^3^Centro de Investigación Biomédica en Red de Fisiopatología de la Obesidad y la Nutrición (CIBEROBN), Instituto de Salud Carlos III, Madrid, Spain

**Keywords:** differentiation, BAT recruitment, preadipocyte, obesity, catecholamine, inflammation, oxidative stress, endoplasmic reticulum stress

## Abstract

Brown adipose tissue (BAT) dissipates energy to produce heat. Thus, it has the potential to regulate body temperature by thermogenesis. For the last decade, BAT has been in the spotlight due to its rediscovery in adult humans. This is evidenced by over a hundred clinical trials that are currently registered to target BAT as a therapeutic tool in the treatment of metabolic diseases, such as obesity or diabetes. The goal of most of these trials is to activate the BAT thermogenic program via several approaches such as adrenergic stimulation, natriuretic peptides, retinoids, capsinoids, thyroid hormones, or glucocorticoids. However, the impact of BAT activation on total body energy consumption and the potential effect on weight loss is still limited. Other studies have focused on increasing the mass of thermogenic BAT. This can be relevant in obesity, where the activity and abundance of BAT have been shown to be drastically reduced. The aim of this review is to describe pathological processes associated with obesity that may influence the correct differentiation of BAT, such as catecholamine resistance, inflammation, oxidative stress, and endoplasmic reticulum stress. This will shed light on the thermogenic potential of BAT as a therapeutic approach to target obesity-induced metabolic diseases.

## Brown Adipose Tissue and Obesity

In recent years, adipose tissue has become a central focus of studies on the mechanisms involved in obesity-related diseases. In humans, this tissue is composed mainly of white adipose tissue (WAT), which stores energy in the form of triglycerides, and brown adipose tissue (BAT), which is responsible for thermogenesis. Much has been learned in the past decades about the pathophysiology of obesity in relation to WAT molecular deregulation (Gesta et al., 2007; Shoelson et al., 2007; de Heredia et al., 2012). However, little was known about these processes in BAT during obesity progression (Villarroya et al., 2018). With the rediscovery of BAT in 2009, and the fact that its mass and activity is reduced in obese and diabetic patients, a door opened for the treatment of obesity and its associated disorders (Cypess et al., 2009; Saito et al., 2009; van Marken Lichtenbelt et al., 2009; Virtanen et al., 2009; Zingaretti et al., 2009).

BAT mass and activity also change with age. In newborns, an increased BAT mass at birth has been related to decreased body fat accumulation during the first 6 months of life (Entringer et al., 2017). In adulthood, a decline of BAT mass and activity has been observed in males and females with age, and may have an impact on the accumulation of body fat (Pfannenberg et al., 2010; Yoneshiro et al., 2011).

Although a few studies have described some characteristics of BAT in a mouse model of diet-induced obesity, the molecular mechanisms involved remain unclear. Recently, it was demonstrated that BAT from obese and hyperglycemic mice shows higher levels of inflammation (macrophages and T cell infiltration), endoplasmic reticulum (ER) stress, oxidative damage, and enhanced mitochondrial respiration activity (Calderon-Dominguez et al., 2016; Alcala et al., 2017). The results of transcriptomic studies have reported several BAT molecular networks modulated in a time-dependent mode in response to a high-fat diet (HFD). The molecular networks are associated with skeletal muscle development, regulation of ion transport, neurotransmitter secretion, the immune system, and lipid metabolism (McGregor et al., 2013; Cao et al., 2018). In addition, several microRNAs (miR) have been identified (miR-491, miR-455, miR-423-5p, miR-132-3p, miR-365-3p, and miR-30b) in obese BAT and could be novel potential pharmacological targets (Gottmann et al., 2018).

## BAT Origin and Differentiation

Most brown adipocytes originate from precursor mesenchymal stem cells (MSC) in the somites during embryonic development. These somatic multipotent precursor cells are characterized by the expression of certain transcription factors such as myogenic factor 5 (Myf5), paired box protein 7 (Pax7), and engrailed-1 (En1) (Atit et al., 2006; Lepper and Fan, 2010; Sanchez-Gurmaches and Guertin, 2014; Wang and Scherer, 2014; Ishibashi and Seale, 2015). Skeletal myocytes, dorsal dermis, and a subset of white adipocytes in certain fat depots also arise from this lineage. Genetic lineage tracing denotes that a multistage process involves the serial activation and repression of transcription factors, co-activators, co-repressors, and cell-cycle regulatory molecules during brown fat adipogenesis (Tapia et al., 2018).

Although the upstream factors that determine brown fat lineage remain unclear, the developmental origin of classical brown adipocytes has been studied in depth (Carobbio et al., 2013; Rosenwald and Wolfrum, 2014). Many transcription factors have been described as core regulators of brown fat development and function, such as peroxisome proliferator-activated receptor γ (PPARγ), CCAAT/enhancer binding proteins (C/EBPα, C/EBPβ, C/EBPδ), PPARγ coactivator 1 alpha (PGC-1α), PRD1-BF1-RIZ1 homologous domain-containing 16 (PRDM16), and even microRNAs. These factors are individually described below.

PPARγ has been described as a master transcription factor in the general differentiation program of brown adipocytes and induces uncoupling protein 1 (*Ucp1)* expression during adipogenesis (Tontonoz and Spiegelman, 2008). Adipocyte-specific *Pparγ^-/-^* animals and the identification of mutations in the *Pparγ* gene in lipodystrophic patients have verified the key role of PPARγ in adipogenesis *in vivo* (Barak et al., 1999; Rosen et al., 1999; Agarwal and Garg, 2002; He et al., 2003). The second group of essential adipogenic transcription factors is the CCAAT/enhancer binding proteins (C/EBPs) (Lane et al., 1999). These transcription factors control the differentiation of a range of cell types and are expressed in early adipogenesis. C/EBPβ and C/EBPδ regulate the expression of C/EBPα and PPARγ, which are involved in the last stages of adipogenic differentiation. However, the mechanism that regulates brown cell lineage determination is not completely clear. Neither PPARγ nor C/EBPs are sufficient to induce and complete the brown adipogenic transcriptional program, although they are considered crucial transcription factors in this process (Tapia et al., 2018).

PRDM16 configures a transcriptional complex with C/EBP-β, which controls the cell fate switch from Myf5^+^ cells to brown preadipocytes (Kajimura et al., 2009; Sanchez-Gurmaches and Guertin, 2014). This transcriptional regulator can activate *Pparγ* expression and induce the thermogenic program. PRDM16 was considered critical for embryonic brown fat development (Sanchez-Gurmaches and Guertin, 2014). Nevertheless, recent studies have shown that brown fat appears in the absence of *Prdm16* expression, because of independent activation of *Pparγ* and *C/ebpα/β* genes during brown fat development (Ishibashi and Seale, 2015). Hence, the participation of PRDM16 in early brown adipogenesis remains unclear.

PGC-1α was initially characterized as a cold-inducible co-activator of PPARγ (Puigserver et al., 1998). This transcription factor was indicated as a key regulator of mitochondrial biogenesis and adaptive thermogenesis. However, the expression of several brown fat-selective genes and the mass of BAT are not affected by genetic ablation of PGC-1α. Hence, PGC-1α does not determine the cellular specification of BAT (Sharma et al., 2014). There has been increasing interest in the differentiation of brown adipocyte by non-coding RNAs (Zhou and Li, 2014; Kajimura et al., 2015). Certain miRs, including miR-378, miR-30, and miR-26, induce brown or beige adipocyte differentiation (Karbiener et al., 2014; Pan et al., 2014; Hu et al., 2015).

Recently, the helix-loop-helix transcription factor, early B-cell factor 2 (EBF2), has been described as an essential mediator of brown adipocyte commitment and terminal differentiation (Rajakumari et al., 2013; Wang W. et al., 2014; Stine et al., 2016; Shapira et al., 2017). EBF2 has been proposed as one of the initial markers in the embryological development of brown fat cells. Moreover, this transcription factor is essential for adequate binding of PPARγ to *Ucp1* and other thermogenic genes (Rajakumari et al., 2013). However, the mechanism by which EBF2 activates the brown fat transcriptional program remains poorly defined.

Finally, bone morphogenetic protein 7 (BMP7) is a new critical candidate for progenitor cells to commit to brown fat lineage (Tseng et al., 2008; Schulz et al., 2013; Chen and Yu, 2018). BMP7 is expressed during the early phase of adipogenesis, and several studies revealed that it induces expression of the early regulator of brown fat fate *Pgc-1α, Prdm16* (Puigserver et al., 1998), as well as the brown adipocyte-specific genes *Ucp1, Dio2, Cidea, Zic1, Tfam*, and *Nrf-1 in vivo* and *in vitro* (Chen and Yu, 2018). Although other BMP family members can enhance adipogenesis *in vitro*, only BMP7 initiates the brown adipogenic program. In fact, MSC fate depends on levels of BMP7 (Tseng et al., 2008). A multifunctional protein EWS (Ewing sarcoma), coupled with its binding partner Y-box binding protein 1 (YBX1), induces *Bmp7* transcription. These results indicate that EWS is also essential for early brown adipocyte lineage determination (Park et al., 2013).

## BAT Quantification in Humans

An increase in BAT mass could emerge as a promising strategy against obesity and related metabolic diseases. Approaches could entail increasing the mass of active cells by promoting differentiation and proliferation or reducing apoptosis of precursor cells.

Prior to the analysis of factors that regulate brown adipocyte recruitment in obese patients, it is important to note that we lack imaging techniques to unequivocally detect the presence of BAT in humans. The traditional combination of ^18^F-fluorodeoxyglucose and computed tomography (^18^F-FDG PET-CT) allows visualization of the tissue from a functional perspective, since the technique is based on detecting radioactive-labeled glucose uptake by the active tissue. Other imaging techniques, recently reviewed in Ong et al. (2018), are also based on functional studies. This implies that patients classified as BAT-negative could be better defined as BAT-inactive. However, the question of whether increased BAT detection is related to preexisting BAT activation or enhanced BAT recruitment still needs to be addressed. Other techniques, such as magnetic resonance imaging (MRI) (Deng et al., 2018) or Xenon-CT (Branca et al., 2018) should be further explored (Sampath et al., 2016). MRI may have benefits over the classic PET-CT approach. For instance, PET-CT is ethically limited in the pediatric population due to ionizing radiation. In addition, the uptake of FDG might be accidentally modified by the room temperature or anesthesia. MRI measurements depend on the hydration state of BAT, which in an obese state is similar to that of WAT and presents high intra- and inter-individual variability (Hu et al., 2013). Inert lipophilic xenon gas in Xenon-CT specifically detects BAT with a high resolution regardless of its activation state. However, it requires the use of ionizing radiation and the implementation of xenon inhalation protocols for its use in humans. Finally, validation of these new imaging methods requires the use of larger cohorts of patients to assess specificity and sensitivity.

## Mechanisms Involved in BAT Expansion

### Cold/Adrenergic Stimulation

Cold-induced adrenergic stimulation is the best-studied intervention for activating the thermogenic program of BAT. Noradrenaline release stimulates UCP-1 expression and WAT lipolysis, which, together with glucose, supplies BAT with energy-rich substrates that are easily oxidable. KO mice models lacking key genes of BAT lipolysis have been used recently to demonstrate that cold-induced thermogenesis requires WAT lipolysis rather than BAT lipolysis (Cannon and Nedergaard, 2017; Schreiber et al., 2017; Shin et al., 2017).

Coupled with this observation, an increase in brown adipocyte recruitment has been reported in rodents since the 1960s (Cameron and Smith, 1964) and more recently suggested in humans. Mild exposure to cold in humans (10°C, 2 h daily for 4 weeks or 15°C, 6 h daily for 10 days) increases the volume of active tissue as reported by ^18^F-FDG PET-CT (van der Lans et al., 2013; Blondin et al., 2014). Even in patients with non-detectable BAT prior to cold intervention, mild exposure to 17°C, 2 h daily for 6 weeks was enough to increase 2-deoxyglucose uptake and BAT activity (Yoneshiro et al., 2013). A similar result was found in a pilot study on young obese patients (Hanssen et al., 2016), although the report has two major drawbacks: a low number of subjects (*n* = 5) and BAT activity was measured rather than BAT volume, as discussed above.

The molecular mechanism of cold-induced BAT recruitment has been thoroughly reviewed (Nedergaard et al., 2018). Briefly, the authors summarize how BAT recruitment in rodents exposed to cold is due to enhanced proliferation of a group of MSC within the tissue, in addition to a reduction in apoptosis (Lee et al., 2015). Unlike white subcutaneous adipocytes, which can activate a thermogenic program by sensing cold without the strict action of adrenergic stimuli (Ye et al., 2013), brown adipocyte proliferation is exclusively linked to the presence of β1 adrenoreceptors, which is the only subtype that is expressed in brown preadipocytes (Bronnikov et al., 1999). In addition, mature adipocyte proliferation can be stimulated by β3 agonists (Fukano et al., 2016). Intracellular signaling involves activation of the cAMP pathway, mediated by well-known mitogen regulators such as phosphatidylinositol-3-kinase (PI_3_K) (Hinoi et al., 2014), mammalian target of rapamycin (mTOR) (Labbe et al., 2016), and extracellular signal-regulated kinase (ERK1/2) (Fredriksson and Nedergaard, 2002). However, *in vitro*, the addition of inhibitors of these pathways was unable to completely inhibit cAMP-mediated cell proliferation (Wang Y. et al., 2014).

Another interesting field of research is how cold affects brown preadipocyte differentiation. In a recent report, the authors describe an *in vitro* model to enhance brown adipocyte differentiation from an immortalized line of mouse MSC by reducing the incubation temperature from 37 to 32°C for 9 days (Velickovic et al., 2018). This could indicate that differentiation can be independent of adrenergic stimulation. However, in this model, differentiated cold-induced cells resemble a beige phenotype according to the expression levels of several beige/brown feature transcription factors.

The key role of adrenergic innervation to enhance BAT recruitment is potentially one of the reasons for the reduced amount of BAT in obese patients. Central obesity has been inversely related to plasma catecholamine levels (Wang et al., 2011). In addition, obesity is characterized by a catecholamine-resistant state, at least in WAT, with reduced expression of adrenergic receptors and a reduced response to noradrenaline-induced lipolysis (Arner, 1999; Guo et al., 2014). We suggest that this situation could also affect BAT, by hampering brown adipocyte proliferation and differentiation.

### Adipokines/Batokines

Immune cells and inflammatory cytokines play a key role in regulation of the thermogenic program of BAT, although knowledge of this process is not as well-known as in WAT. Some of the latest evidence has been thoughtfully reviewed in van den Berg et al. (2017). For instance, alternatively activated M2 macrophages have been reported to be necessary to sustain a cold-adaptive thermogenic program in BAT, probably due to the ability to synthesize catecholamines (Nguyen et al., 2011). Regulatory T cells are also required to maintain a proper adaptive response to cold. Genetic ablation of this type of immune cells impaired the expression of thermogenic markers and promoted the invasion of proinflammatory macrophages (Medrikova et al., 2015).

In obesity, BAT shows a low-degree of inflammation characterized by the M1 macrophage, T cell infiltration, regulatory T cell decline and cytokine release. However, it takes longer to appear and has a more limited extension than in white adipose depots (Fitzgibbons et al., 2011; Alcala et al., 2017). Time-course microarrays on HFD-fed mice revealed that the upregulation of immune cell trafficking genes begins after week 8 and spikes by week 20, together with an inflammatory response (McGregor et al., 2013). The infiltration of M1 macrophages and the proinflammatory cytokines that are released promotes a decline in UCP-1 expression, which alters thermogenic activity (Sakamoto et al., 2016).

In addition, infiltrated immune cells with pro-inflammatory potential and both circulating and self-synthesized chemokines can inhibit BAT recruitment during obesity. During normal brown preadipocyte differentiation, there is a time-dependent downregulation of the expression of pattern recognition receptors such as NOD2 and TLR2, both upstream of the NF-kB proinflammatory pathway (Bae et al., 2014). When these receptors are activated by their corresponding agonists, brown preadipocyte differentiation and adipogenesis are inhibited in a NF-κB-dependent mechanism (Bae et al., 2015). Similarly, exposure to pro-inflammatory molecules such as TNF-α, IL-1, LPS, or Oncostatin M, secreted by T cells and macrophages, inhibits brown differentiation *in vitro* (Mracek et al., 2004; Zoller et al., 2016; Sanchez-Infantes et al., 2017). This is achieved by downregulating key adipogenic factors such as PPARγ, which reproduces effects that were previously observed in white preadipocytes (Ron et al., 1992).

Inflammatory signals can also promote cellular apoptosis, which impedes the expansion of BAT. For instance, the induction of apoptosis by TNF-α has been traditionally described in white (Furuoka et al., 2016; Zoller et al., 2016) and brown adipocytes (Valladares et al., 2000; Miranda et al., 2010).

Finally, inflammation can inhibit brown adipocyte proliferation indirectly by inhibiting catecholamine signaling (Villarroya et al., 2018). As mentioned above, noradrenaline promotes brown adipocyte proliferation and preadipocyte differentiation. However, obesity-induced inflammation may reduce the noradrenergic tone by several potential mechanisms:

(1) Interrupting cAMP intracellular signaling. IKK𝜀 overexpression drives the activation of NF-κB and phosphodiesterases, which reduces the availability of cAMP (Mowers et al., 2013).
(2) Reducing the synthesis of catecholamines. Traditionally, it was claimed that the obesity-induced phenotype shift from M2 anti-inflammatory macrophage to M1 pro-inflammatory macrophage was accompanied by loss of the capacity to express tyrosine hydroxylase (Nguyen et al., 2011), the rate-limiting step in the synthesis of noradrenaline. However, recent studies question the initial capacity of M2 macrophages to express TH (Fischer et al., 2017).
(3) Enhancing the clearance of catecholamines. Growth differentiation factor-3 (GDF3), a member of the TGF-β family, enhances the activity of monoamine oxidase in macrophages through activation of the inflammasome system. This promotes noradrenaline uptake and degradation (Guo et al., 2014).

### Oxidative Stress

During obesity development, mitochondrial dysfunction due to increased substrate oxidation, together with the action of other oxidases, increases the production of reactive oxygen species (ROS). As a compensatory mechanism, the expression of antioxidant enzymes is upregulated via NRF2 and FOXO, which maintains the intracellular redox state. Oxidative stress occurs when excessive production of ROS overrides the antioxidant defense, causing macromolecule oxidation. In WAT, oxidative stress is one of the mechanisms that accounts for malfunction of the adipocyte, since it has an impact on insulin signaling or inflammation, among other factors (Furukawa et al., 2004; Jankovic et al., 2014; Alcala et al., 2015). Obese mouse BAT presented the same signs of oxidative stress: increased ROS production and a decline in antioxidant capacity (Alcala et al., 2017).

The relevant role of oxidative stress in BAT recruitment lies in the dual effect of ROS as pro-oxidative molecules at high pathological concentrations when they surpass the antioxidant defense, and their action as second messengers in cell signaling processes at physiological levels (Jones and Sies, 2015; Castro et al., 2016).

Unfortunately, little is known about the effect of ROS on BAT differentiation. A recent report describes an increase in mRNA and protein levels of antioxidant enzymes during murine brown preadipocyte differentiation (Rebiger et al., 2016). The same was observed in white adipocytes, in which the expression of antioxidant enzymes during differentiation is also increased to prevent oxidative stress (Adachi et al., 2009; Higuchi et al., 2013). In fact, Furukawa et al. (2004) described for the first time that *in vitro* differentiation of 3T3-L1 adipocytes was accompanied by an increase in ROS formation. These results have been reproduced in other *in vitro* models of white preadipocytes (Kanda et al., 2011). The addition of extracellular H_2_O_2_ shows a concentration-dependent effect on differentiation in the micromolar range. This enhances adipogenesis by acting on PPARγ and the C/EBP family of transcription factors (Tormos et al., 2011; Higuchi et al., 2013) to accommodate the excess of fat and accelerating cell proliferation (Lee et al., 2009).

However, to the best of our knowledge, there is little evidence of the role of ROS in the regulation of differentiation and adipogenesis in BAT, although some reports point to the role of Wnt signaling. BAT expresses Wnt10a, which is upregulated, at least *in vitro*, by the addition of H_2_O_2_ (Yasuniwa et al., 2010) and Wnt10b. Wnt activation leads to impaired brown preadipocyte differentiation and whitening of the mature brown adipocyte (Kang et al., 2005).

The incubation of preadipocytes with antioxidants led to reduced ROS formation and impaired differentiation (Calzadilla et al., 2011; Hou et al., 2012), which reflects the need to maintain a proper intracellular redox balance. Similarly, when BSO, a glutathione quencher, was added to mimic an oxidative stress situation, ROS production increased but differentiation was inhibited (Findeisen et al., 2011).

If these molecular pathways were common between white and brown preadipocytes, they would lead to changes in BAT phenotype during obesity. Changes would range from “whitening” as ROS secretion begins to a complete loss of the ability to differentiate new cells when oxidative stress is established. As a result, thermogenic capability would be completely lost.

### Endoplasmic Reticulum Stress

During obesity, the overload of protein folding requirement can trigger ER stress to activate the unfolded protein response (UPR). Briefly, the UPR tries to restore the function of the ER through three pathways: decreasing protein translation, enhancing protein folding, and triggering cellular apoptosis if the repair process fails. Key markers of UPR such as *Bip, Chop, Atf4*, or *Atf6* expression have been found to be overexpressed in BAT from HFD-fed obese mice (Alcala et al., 2017; Liu et al., 2017). However, caspase 3 was not overexpressed, which indicates that the UPR was activated for a reparative rather than a proapoptotic end. Actually, when HFD-fed mice were further induced ER stress with the administration of thapsigargin or tunicamycin, gene expression of caspase 3 and 12 and *Bax* was upregulated (Liu et al., 2017).

A recent report uses two approaches to inhibit UPR in brown preadipocytes: incubation with 4-phenyl butyric acid (4-PBA), an inhibitor of ER stress, and siRNA for Xbp1. Both approaches drastically reduced differentiation and adipogenesis, which indicates the key role of the activation of UPR during differentiation. Furthermore, the incubation of brown preadipocytes with capsaicin, a component of red chili peppers, stimulates brown adipogenesis as well as the expression of UPR genes such as Xbp1 or Chop (Kida et al., 2018). More recently, Ju et al. (2018) described the role of a member of the Bcl2 family, Bcl2l13, in the differentiation of brown preadipocytes. Bcl2l13 play a key role in the regulation of mitochondrial dynamics. Silencing Bcl2l13 expression resulted in hampered brown preadipocyte differentiation due to decreased expression of mitochondrial fusion genes, biogenesis and respiratory chain complexes.

These results may suggest that reparative branches of the UPR must be activated for brown preadipocyte differentiation, in a similar manner to that previously observed in white differentiation (Sha et al., 2009). However, when ER stress is strongly induced (by chemical inducers or by long-term obesity), the apoptotic pathway can be triggered, which participates partially in BAT atrophy. BAT from obese rats has lower Bcl-2/Bax mRNA and protein ratios than the BAT of their lean littermates (Briscini et al., 1998).

### miRNA

miRNAs are a type of single-stranded mRNA with a variable size ranging from 21 to 25 nucleotides. They play a key role in the regulation of gene expression, mainly by binding to the 3′UTR of target mRNAs and blocking their translation. In the last few years, the role of several miRNAs in adipose tissue biology has been revealed (Brandao et al., 2017). More specifically, miRNA arrays were used to detect at least 25 BAT-enriched miRNA genes targeting up to 788 genes involved in brown adipocyte growth, proliferation and differentiation (Guller et al., 2015; Price and Fernandez-Hernando, 2016). However, the regulation of these miRNAs in obesity has not yet been determined. Findings regarding miRNAs and BAT expansion in obesity are summarized in Table 1.

**Table 1:** miRNA involved in BAT expansion.

miRNA	Effect in differentiation	Target gene	Obesity	Reference
miR-27	↓	Prdm16 Creb Pparα Pgc-1β	miR-27 expression correlates with BMI	Yu et al., 2018 Sun and Trajkovski, 2014
miR-34a	↓	Fgf21 Sirt1	miR-34a expression is increased in obesity. miR-34a overexpression protected against HFD-induced obesity	Fu et al., 2014
miR-133	↓	Prdm16	Antagonist miR-133 treatment protected against HFD-induced obesity	Yin et al., 2013
miR-155	↓	Cebpβ	miR-155 KO protected against HFD-induced obesity	Gaudet et al., 2016
miR-199a-214	↓	Prdm16 Pgc-1α	miR-199a-214 expression reduced in genetic models of obesity. Anti-miR-199a-214 injection protected against body weight gain	He et al., 2018
miR-328	↑	Bace1	HFD-induced obesity increased miRNA-328 expression in BAT	Oliverio et al., 2016
miR-378	↑	Pde1b	miR-378 promoted BAT expansion, protecting against genetic and HFD-induced obesity	Pan et al., 2014
miR-455	↑	Runxlt1 Necdin	miRNA-455 transgenic mice (FAT455) protected against HFD-induced obesity	Zhang et al., 2015
miR-93-106b	↓	Ppparα	HFD-induced obesity increased miRNA-93-106b expression in BAT	Wu et al., 2013

*Role in obesity.*

## Conclusion

Although not completely understood yet, brown adipocyte differentiation involves a complex network of transcription factors, genes, and miRNAs that are apparently interrupted in obesity.

**FIGURE 1:**
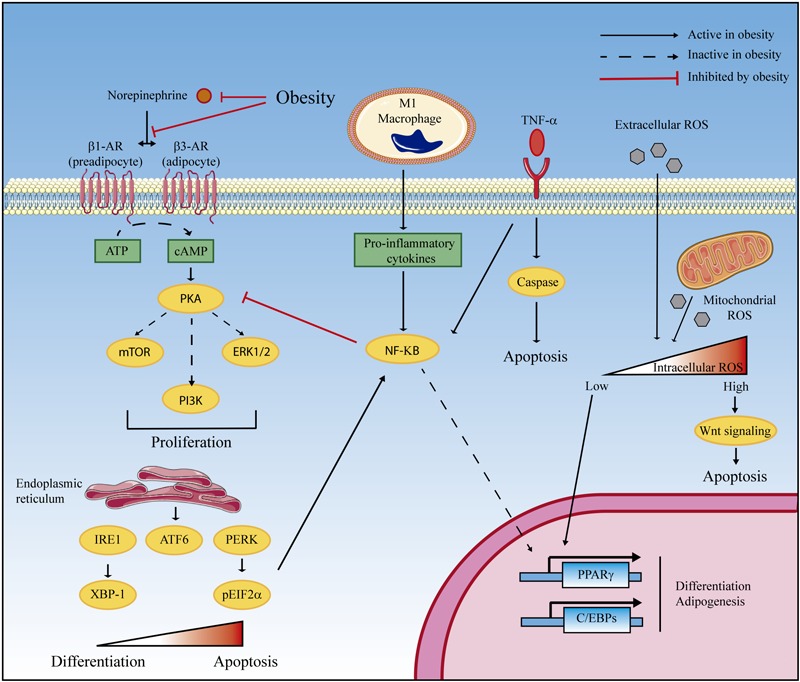
Proposed mechanisms of obesity-induced BAT depletion. BAT mass and activity are minimized in obese patients due to reduced cell proliferation and preadipocyte differentiation and increased apoptosis. At least four obesity-related mechanisms can be involved. (1) Catecholamine resistance in obesity is characterized by decreased synthesis of norepinephrine and beta-adrenergic (β-AR) receptors and by defective intracellular signaling, which impedes PKA-mediated cell proliferation. (2) Obesity promotes the infiltration of M1 macrophages that participate in norepinephrine clearance and contribute to the synthesis of proinflammatory cytokines. NF-κB-mediated signaling inhibits the PKA proliferation pathway and represses PPARγ and C/EBPs gene expression, which inhibits differentiation and adipogenesis. In addition, TNF-α overexpression triggers cellular apoptosis. (3) The unfolded protein response (UPR) in the endoplasmic reticulum plays a dual role in brown adipocyte differentiation according to the intensity of the signal. While activation of the three branches of UPR (IRE-1, ATF6, and PERK) is required for differentiation, excessive UPR activation (that can be found in severe obesity) triggers proapoptotic mechanisms. (4) In a similar manner, reactive oxygen species (ROS) are also hormetic regulators of cell differentiation and apoptosis. Physiological ROS concentrations promote C/EBP expression leading to differentiation, whereas supraphysiological concentration leads to oxidative stress and apoptosis via Wnt signaling. Artwork was obtained from Servier Medical Art, licensed under a Creative Common Attribution 3.0 Generic License (http://smart.servier.com/).

The relations between the pathological basis of obesity and strategies to recruit active BAT are summarized in Figure 1. Resistance to catecholamines and inflammatory processes directly reduce adipocyte differentiation and proliferation and can promote apoptosis. In addition, a moderate increase in ROS generation (before antioxidant defense is surpassed and oxidative stress is established) and ER stress favors brown preadipocyte differentiation and adipogenesis. The goal would most likely be to accumulate an excess of fat, which involves brown adipocytes changing to acquire a white phenotype, and thus losing their thermogenic potential. If the obesity challenge continues and oxidative stress and ER stress are completely established, both differentiation and proliferation are inhibited, and cellular apoptosis is triggered. The decrease of BAT mass and activity in obese individuals indicates that any strategy leading to an enhancement of preadipocyte differentiation and proliferation or a reduction of apoptosis could be potentially added to the therapeutic arsenal against obesity.

## Author Contributions

MA, MC-D, and MV participated in the conception of the study and in the preparation of the manuscript. LH and DS participated in the preparation of the manuscript and critically reviewed the final draft.

## Conflict of Interest Statement

The authors declare that the research was conducted in the absence of any commercial or financial relationships that could be construed as a potential conflict of interest.

## Abbreviations

4-PBA: 4-phenyl butyric acid
ATF: Activating transcription factor
BAT: Brown adipose tissue
BIP: Binding immunoglobulin protein
BMP7: Bone morphogenic protein 7
C/EBP: CCAAT-enhancer-binding proteins
CHOP: C/EBP homologous protein
EBF2: Early B cell factor 2
EN1: Homeobox protein engrailed 1
ER: Endoplasmic reticulum
ERK: Extracellular signal-regulated kinase
EWS: Ewing sarcoma protein
FOXO: (Forkhead box) proteins
GDF-3: Growth differentiation factor 3
HFD: High-fat diet
IL-1: Interleukin 1
LPS: Lipopolysaccharide
MSC: Mesenchymal cells
mTOR: Mammalian target of rapamycin
MYF5: Myogenic factor 5
NF-κB: Nuclear factor kappa B
NOD: Nucleotide-binding oligomerization domain-containing protein
NRF2: Nuclear factor (erythroid-derived 2)-like 2
PAX7: Paired box-protein 7
PI3K: Phosphatidylinositol-3-kinase
PPAR: Peroxisome proliferator-activated receptor
PRDM16: PR domain containing 16
TLR: Toll-like receptor
TNF-α: Tumor necrosis factor Alpha
UCP1: Uncoupling protein 1
UPR: Unfolded protein response
WAT: White adipose tissue
XBP1: X-box binding protein 1
YBX1: Y-box binding protein 1
